# A Case of Bilateral Congenital Pseudoarthrosis of Clavicle: An Uncommon Variant of a Rare Disorder

**DOI:** 10.7759/cureus.66594

**Published:** 2024-08-10

**Authors:** Ahmad N Boeisa, Alya A Alshammary, Sara Albunyan, Lina AlMudayris, Mohammed AlSaeed

**Affiliations:** 1 Pediatric Orthopaedics, Almoosa Specialist Hospital, Al-Mubarraz, SAU; 2 College of Medicine, King Faisal University, Al-Hofuf, SAU; 3 Orthopaedics, King Fahad Hofuf Hospital, Al-Hofuf, SAU

**Keywords:** fracture, congenital deformity, clavicle, pediatric orthopedics, congenital pseudarthrosis of the clavicle

## Abstract

Congenital pseudarthrosis of the clavicle (CPC) is a rare disorder diagnosed at birth or early childhood presenting with a painless, non-tender mass on the clavicle. Its etiology is unknown, caused by failure of fusion of the medial and lateral ossification centers of the clavicle. Left-sided CPC is rare and linked to other pathological abnormalities. Bilateral involvement is extremely rare and it is seen in association with other congenital malformations. A full-term newborn baby girl was examined after a complicated emergency cesarean section delivery. Upon initial pediatric examination, there was suspicion of bilateral clavicle fracture with no limitation of movement and equal moro reflex bilateral. Plain radiographs of the clavicle revealed a suspected bilateral fracture of the clavicle. At the two-month follow-up, X-rays were taken to assess the clavicle fractures showing persistent bilateral clavicle deformities and there was no interval callus formation which confirmed the diagnosis of bilateral CPC and excluded the presence of the fracture. Bilateral pseudarthrosis of the clavicle is a rare entity, and surgical correction is not required unless the patient develops symptoms of limitations of movement or for aesthetic causes.

## Introduction

Congenital pseudarthrosis of the clavicle (CPC) is a rare entity diagnosed at birth or early childhood. Its etiology is unknown, but theories suggest that it is triggered by the failure fusion of the two ossification nuclei of the clavicle in utero during embryogenesis [1-2].

Right-sided CPC is more common and is mostly seen in girls. Left CPC is unusual and is commonly linked to other pathologies such as dextrocardia, situs inversus, and cervical ribs. Bilateral involvement is very rare; it may occur in less than 10% of the cases, and it is seen in association with other congenital malformations, e.g., trisomy 22. It is caused by the presence of cervical ribs or vertical turning of the upper ribs, causing an abnormally high position of the subclavian artery [3-5].

The diagnosis of CPC in a newborn is made through a comparison of the first plain radiographs in which a fracture of the clavicle may be suspected with another image later on showing no interval callus formation, which will confirm the diagnosis of CPC [5]. The mainstay of the treatment is observation, with no treatment required. However, CPC may require surgical correction that can be done at a later age. It is indicated for cosmetic reasons and once symptoms affect patients' lives [6].

## Case presentation

A full-term female infant, weighing 3.5 kg, was delivered via emergency cesarean section after a gestation period of 39 weeks and five days. Upon delivery, the infant manifested a concerning clinical presentation characterized by a flat airway and cyanosis. Immediate resuscitation measures were instituted, commencing with placement under a warmer for 30 seconds, followed by a systematic evaluation of vital signs. The infant exhibited bradycardia (60/minutes) persisting despite initial resuscitative efforts. Positive pressure ventilation (PPV) with an Ambu bag was initiated for 15 seconds, resulting in an improvement in heart rate to 120/minutes. However, persistent apnea and cyanosis prompted the decision to proceed with endotracheal intubation using an endotracheal tube (ETT) sized 3.5 mm at a depth of 9.5 cm. Proper placement was confirmed, and the tube was secured.

After intubation, the infant's heart rate increased to 150/minute, accompanied by irregular respirations and a transition to a pink coloration. After 10 minutes, the infant demonstrated regular breathing, fair activity, spontaneous movements, and sustained pink coloration. Following successful stabilization, extubation was performed, and the infant was transitioned to a nasal cannula at 3 L/minutes.

**Figure 1:**
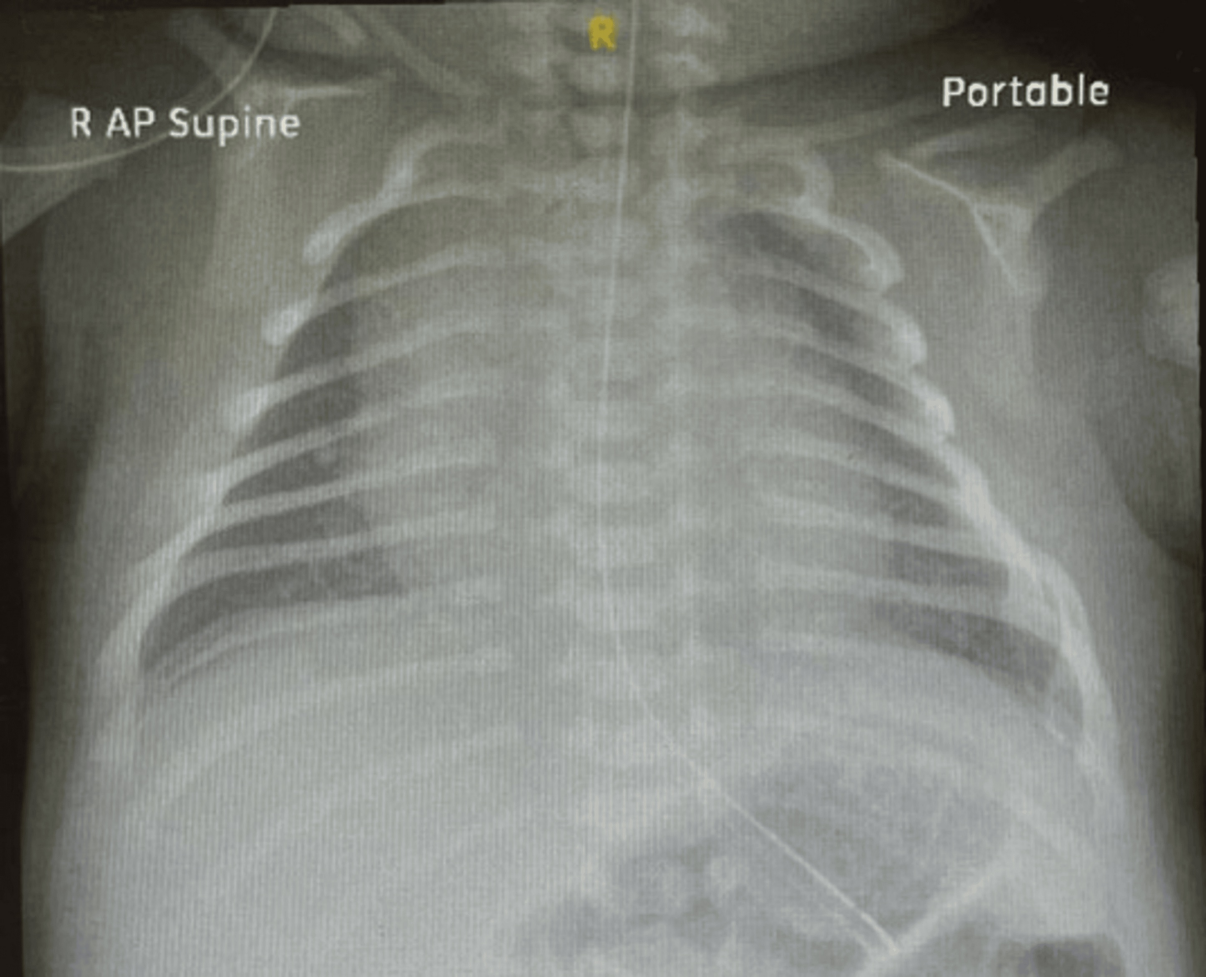
Initial Bilateral Clavicle X-ray.

**Figure 2:**
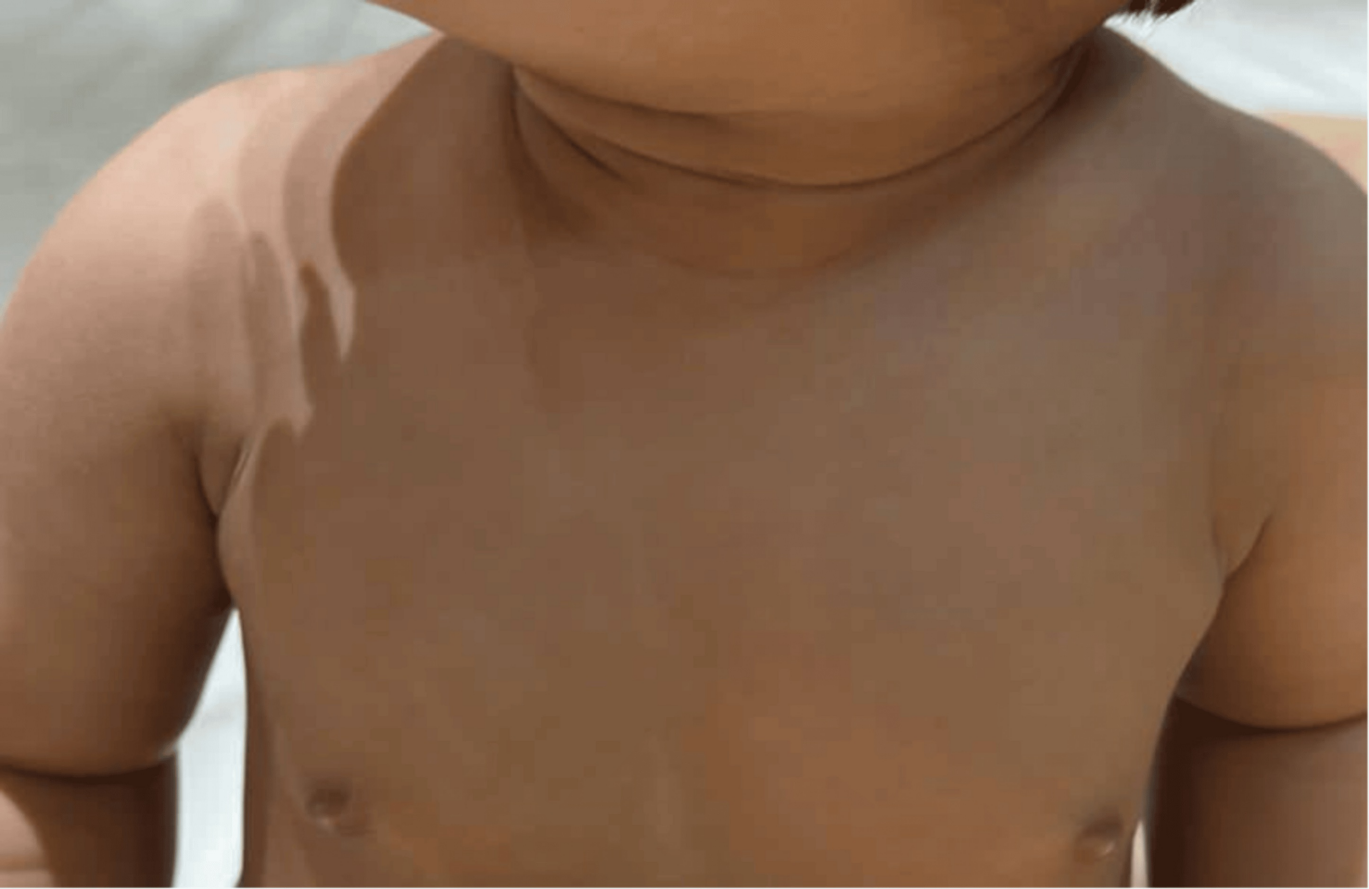
Clinical Picture of the Patient in Out Patient Department Follow-Up.

**Figure 3:**
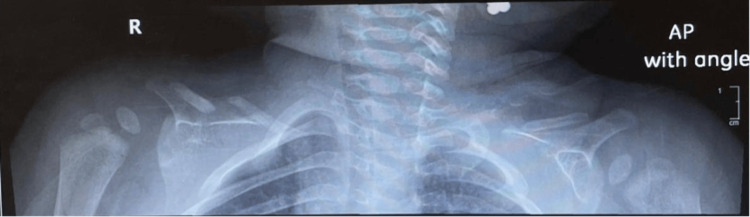
Bilateral Clavicle X-ray During Out Patient Department Follow-Up.

Given the clinical presentation, a suspicion of bilateral clavicle fracture emerged, prompting a referral for further evaluation. Subsequent X-ray examination of the clavicles confirmed the diagnosis of bilateral clavicular fractures (Figure 1). At one month of age, the infant presented for follow-up with the orthopedic department post discharge. The patient had no obvious deformities in both of the clavicles. Also, there was no palpable swelling over both clavicles with no tenderness upon palpation. However, discontinuity of the clavicle shaft was noted by palpation. Shoulders displayed a full range of movement with no limitations or audible sounds (Figure 2). Repeated X-rays indicated no healing of clavicular shaft fractures, with pseudoarthrosis of the clavicle bilaterally (Figure 3). The parents were reassured that this was not a fracture and informed that this was a rare congenital disorder with no need for any modality of treatment other than observation as the patient was asymptomatic with no functional limitations. 

## Discussion

CPC is an uncommon condition usually identified within the initial years of life, resulting from the inability of the ossification nuclei of the clavicle to fuse appropriately during intrauterine development [7]. The abnormality is commonly found on one side and is situated in the central portion of the clavicle [8]. While CPC itself is considered rare, the occurrence of bilateral involvement is exceptionally unusual and occurs in only 10% of cases [7]. This case report details a rare presentation of bilateral CPC, adding to the limited literature on this variant of CPC.

Pseudarthrosis of the clavicle typically manifests as a painless bulge on the clavicle, frequently occurring in the central or lateral third of the bone. Beyond its visual impact, pseudarthrosis generally lacks symptoms. Nevertheless, certain individuals may encounter pain, discomfort, or functional constraints, potentially leading to conditions like late-onset thoracic outlet syndrome [9]. From a radiographic perspective, a diagnosis is typically achievable with plain films when the clinical history and physical examination align [10]. Typically, these cases involve the middle section of the clavicle, showing a clear division into two parts. The absence of callus formation at the pseudarthrosis site, coupled with an uncomplicated delivery, aids in distinguishing it from the more prevalent alternative diagnosis of clavicular fracture with nonunion [11]. In our case, diagnosing bilateral CPC presented a considerable dilemma due to several factors. Initially, the infant's clinical presentation included a complex set of symptoms such as flat apnea, cyanosis, and low APGAR scores, which prompted immediate resuscitation efforts. These critical conditions necessitated a focus on addressing the immediate life-threatening concerns, temporarily diverting attention from musculoskeletal considerations. Moreover, the rarity of bilateral CPC added a layer of complexity to the diagnostic challenge. The condition itself is uncommon, and bilateral involvement is atypical, making it an unusual consideration during the initial assessment. The definitive confirmation occurred during the follow-up period, where dedicated musculoskeletal assessments and radiographic examinations provided clarity, allowing for a precise diagnosis and appropriate management decisions.

Due to the infrequency of CPC, there is a limited number of comparative studies assessing surgical versus conservative approaches. The consideration for conservative treatment arises when the child with CPC experiences no pain or activity limitations [12]. Surgical intervention may be recommended for reasons such as addressing cosmetic concerns, alleviating shoulder drooping, managing discomfort, alleviating pain, addressing functional symptoms, or preventing complications like thoracic outlet syndrome and other vascular pathologies [13]. Surgical treatment comprises the removal of pseudoarthrosis, grafting of bone, and internal fixation [12]. A study documented a distinctive instance involving a newborn diagnosed with bilateral CPC in which the segments of the clavicle spontaneously fused, exhibiting normal size and configuration in both clavicles [14]. In our patient's case, a decision was made to adopt a conservative approach by opting for follow-up and monitoring without immediate surgical intervention. Given the rarity of CPC and the infant's relatively stable clinical presentation, the decision to refrain from surgical intervention was guided by the absence of immediate indications for intervention, such as significant pain or functional impairment. By opting for a watchful waiting approach, the medical team aimed to assess the natural progression of the bilateral CPC over time, allowing for a more informed decision-making process regarding potential future interventions. This approach also considers the potential for spontaneous resolution or stabilization of the condition without the need for surgical procedures.

## Conclusions

CPC is a rare condition caused by the failure of ossification nuclei to fuse during embryonic development. This case report presents a highly unusual instance of bilateral CPC, contributing to the limited literature on this condition. Diagnosing bilateral CPC can be difficult, especially when the first symptoms require rapid resuscitation.

Conservative observation is the primary management approach, particularly in asymptomatic cases. Patients with severe pain, functional impairment, or cosmetic issues may require surgical intervention. A cautious strategy was selected in the current case due to the patient's stable condition and the lack of immediate surgical indications. This report emphasizes the significance of taking CPC into account while making a differential diagnosis of neonatal clavicular anomalies, as well as the relevance of long-term monitoring. Further research is needed to develop standardized management guidelines for CPC, particularly in bilateral cases.
